# The Effects of Vitamin E from *Elaeis guineensis* (Oil Palm) in a Rat Model of Bone Loss Due to Metabolic Syndrome

**DOI:** 10.3390/ijerph15091828

**Published:** 2018-08-24

**Authors:** Sok Kuan Wong, Kok-Yong Chin, Farihah Hj Suhaimi, Fairus Ahmad, Soelaiman Ima-Nirwana

**Affiliations:** 1 Department of Pharmacology, Faculty of Medicine, Universiti Kebangsaan Malaysia, Jalan Yaakob Latif, Bandar Tun Razak, Cheras 56000, Kuala Lumpur, Malaysia; jocylnwsk@gmail.com (S.K.W.); chinkokyong@ppukm.ukm.edu.my (K.-Y.C.); 2 Department of Anatomy, Faculty of Medicine, Universiti Kebangsaan Malaysia, Jalan Yaakob Latif, Bandar Tun Razak, Cheras 56000, Kuala Lumpur, Malaysia; farihah_suhaimi@yahoo.com (F.H.S.); apai.kie@gmail.com (F.A.)

**Keywords:** dyslipidemia, hyperglycemia, hypertension, obesity, osteoporosis, tocopherol, tocotrienol

## Abstract

The beneficial effects of vitamin E in improving components of MetS or bone loss have been established. This study aimed to investigate the potential of palm vitamin E (PVE) as a single agent, targeting MetS and bone loss concurrently, using a MetS animal model. Twelve-week-old male Wistar rats were divided into five groups. The baseline group was sacrificed upon arrival. The normal group was given standard rat chow. The remaining three groups were fed with high-carbohydrate high-fat (HCHF) diet and treated with tocopherol-stripped corn oil (vehicle), 60 mg/kg or 100 mg/kg PVE. At the end of the study, the rats were evaluated for MetS parameters and bone density. After euthanasia, blood and femurs were harvested for the evaluation of lipid profile, bone histomorphometric analysis, and remodeling markers. PVE improved blood pressure, glycemic status, and lipid profile; increased osteoblast surface, osteoid surface, bone volume, and trabecular thickness, as well as decreased eroded surface and single-labeled surface. Administration of PVE also significantly reduced leptin level in the HCHF rats. PVE is a potential agent in concurrently preventing MetS and protecting bone loss. This may be, in part, achieved by reducing the leptin level and modulating the bone remodeling activity in male rats.

## 1. Introduction

A group of at least three medical conditions, such as obesity, hypertension, hyperglycemia, hypertriglyceridemia, and low high-density lipoprotein (HDL) cholesterol is termed a metabolic syndrome (MetS) [1]. Increasing evidence from human studies suggests a relationship between MetS and osteoporosis, and whether MetS confers positive [2] or negative impact on the bone [3,4]. The heterogeneous outcomes are attributed to the different and independent effects of abdominal obesity, hypertension, hyperglycemia, and dyslipidemia on the skeleton. Interestingly, previous studies demonstrated that MetS was associated with musculoskeletal disorders such as osteoporosis [5,6] and osteoarthritis [7,8]. However, the underlying mechanisms involved in orchestrating the bone metabolism by MetS remains to be seen.

Metabolic syndrome often develops over time, from a sustained positive energy balance and excessive nutrition. Leptin and adiponectin are adipocyte-derived hormones that respond to fat mass, and influence energy intake and expenditure in different ways [9,10,11]. In addition, a hyperglycemic condition is unequivocally linked with hyperinsulinemia to induce glucose uptake and normalize blood glucose. Insulin resistance is characterized by the concurrence of hyperglycemia and hyperinsulinemia during fasting [12]. Therefore, over-nutrition in MetS can disrupt the body energy regulation governed by leptin, adiponectin, and insulin. The receptors of leptin, adiponectin and insulin are expressed in osteoblast, indicating that these hormones play a role in bone metabolism [13,14,15]. Leptin exerts dual controls in bone metabolism. Peripheral leptin improves bone health by increasing estrogen, decreasing cortisol and glucocorticoids [16]. Leptin also acts as a potent inhibitor for bone formation through the central nervous system [17,18]. Adiponectin has been seen to induce osteoblast proliferation and differentiation [19]. In addition, the binding of insulin and its receptors on osteoblast can either stimulate bone formation via insulin receptor substrate (IRS-1 and IRS-2) [15], or increase bone resorption via the reduction of osteoprotegerin (OPG)/receptor activator of nuclear factor kappa-Β ligand (RANKL) ratio [20]. Combined, the imbalance of leptin, adiponectin and insulin caused by MetS may result in the uncoupling of the bone remodeling process, leading to bone loss.

Currently, the first line treatment for MetS focuses on lifestyle modification. Medicines will be prescribed if lifestyle changes are unsuccessful. However, the medicines used usually tackle each medical condition come with undesirable side effects [21]. Pharmacological therapeutic agents such as hormone replacement therapy, bisphosphonates, and teriparatide reverse osteoporosis also come with some adverse side effects [22]. Therefore, the need to find a single agent to target both MetS and osteoporosis, concurrently with minimal side effects, is essential. Tocotrienol is found in vitamin E with four analogues, namely alpha- (α-), beta- (β-), gamma (γ-), and delta-(δ-)tocotrienol. The effects of vitamin E in preventing components of MetS have been well-established [23,24]. Specifically, the anti-obesity [25], anti-hyperglycemic [26], anti-hypertensive [27], anti-hypercholesterolemic [28], anti-oxidative [29], and anti-inflammatory properties [30] of vitamin E have been also reported. Apart from that, vitamin E possesses bone-protective properties in various surgical castrated [31,32] and chemically-induced osteoporotic animal models [33,34]. In addition, our recent data demonstrated that tocotrienol improved MetS features and bone microstructure in male rats [35,36].

This study aimed to further evaluate the effects of palm vitamin E (PVE) in preventing MetS and bone loss with respect to changes in bone mineral density (BMD), bone histomorphometry, and remodeling markers, using a high-carbohydrate, high-fat (HCHF) diet-induced MetS animal model. We also investigated the involvement of hormonal changes (leptin, adiponectin, and insulin) caused by MetS in mediating bone metabolism in male rats. Due to the beneficial properties of PVE, we hope to validate the concurrent use of PVE as a potential single agent for the management of MetS and osteoporosis.

## 2. Materials and Methods

### 2.1. Experimental Design

Animal experimentation protocols were reviewed and approved by the Universiti Kebangsaan Malaysia Animal Ethics Committee (Code: PP/FAR/2015/IMA/20-MAY/679-JUNE-2015-MAY-2017) and the procedures were conducted according to the approved guidelines. Male Wistar rats aged twelve weeks old were supplied by Laboratory Animal Resource Unit, Universiti Kebangsaan Malaysia (Kuala Lumpur, Malaysia). The rats were housed individually for one week at the Animal Laboratory of Department of Anatomy, Universiti Kebangsaan Malaysia (Kuala Lumpur, Malaysia) under standard ambient room temperature (25 ± 2 °C) to adapt. Light and darkness were alternated 12 h apart at the facility. Upon acclimatization, the rats were randomly divided into five experimental groups (*n* = 6/group). The baseline group was sacrificed upon arrival. The normal group was assigned with standard rat chow and tap water. The remaining three groups were fed with HCHF diet (composed of 395 g sweetened condensed milk, 200 g of ghee, 175 g of fructose, 155 g of powdered rat food, 25 g of Hubble Mendel and Wakeman salt mixture, 50 mL of water) and 25% fructose-supplemented drinking water [37]. The estimated nutrient content for standard and HCHF diet has been described elsewhere [6]. The rats were allowed access to food and water *ad libitum*. The HCHF diet-fed animals were treated with tocopherol-stripped corn oil (the vehicle of PVE), 60 mg/kg, or 100 mg/kg PVE. Treatment was administered daily via oral gavage and commenced after feeding the animals with the HCHF diet for eight weeks. The PVE used in this study composed of 21.9% α-tocopherol, 24.7% α-tocotrienol, 4.5% β-tocotrienol, 36.9% γ-tocotrienol, and 12.0% δ-tocotrienol (Batch number: A1/50/0519_1_120315), (Excelvite Sdn Bhd, Chemor, Malaysia). MetS parameters (abdominal circumference, blood pressure, fasting blood glucose, and oral glucose tolerance test), as well as BMD of animals were evaluated once at the end of the 20 week study, before sacrifice. After that, rats were sacrificed with overdoses of ketamine-xylazine combinations (Dose: ketamine, 300 mg/kg; xylazine, 30 mg/kg). Blood and femurs were collected for further analysis.

### 2.2. Metabolic Syndrome Parameters

Food, water and energy intake were measured and calculated daily. The energy densities for standard rat chow, HCHF diet and fructose-supplemented drinking water were 13.80, 17.81, and 3.85 kJ/g respectively. The abdominal circumference of the animals was measured using standard measuring tape, under general anesthesia (Dose: ketamine, 100 mg/kg; xylazine, 10 mg/kg). After warming the rats for 10 min, their systolic and diastolic blood pressure were monitored using the non-invasive tail-cuff system (CODA™, Kent Scientific Corporation, Torrington, CT, USA). For the determination of basal blood glucose concentration, the animals were deprived of all types of food, and fructose drinking water was replaced with tap water for 12 h (overnight). For an oral glucose tolerance test (OGTT), a glucose load (2 g/kg) was given to the animals as 40% glucose solution via oral gavage. Glucose concentrations were then measured after 30, 60, 90, 120 min. The blood glucose levels were determined using the Accu-Check Performa glucose meter (Roche Diagnostic, Indianapolis, IN, USA). The area under the curve (AUC) of integrated blood glucose measurements over time was calculated using the trapezoidal rule. The blood collected upon euthanasia was left to clot and processed (centrifuge at 1800× *g* for 10 min) to obtain serum. Lipid profile (triglyceride, total cholesterol, HDL cholesterol, and LDL cholesterol) was determined using commercial colorimetric assay kits (BioAssay Systems, CA, USA).

### 2.3. Dual-Energy X-ray Absorptiometry (DXA)

Whole body (the assessment of fat mass and whole-body BMD) and regional (the assessment of femoral and tibial BMD) scans were performed under general anesthesia using dual-energy X-ray absorptiometry (DXA) (Hologic QDR-1000 System, Hologic Inc., Waltham, MA, USA). The scans were analyzed using the manufacturer’s recommended software (Small Animal Analysis Software, Hologic QDR-1000 System) to evaluate whole body, femur, and tibia BMD of the animals.

### 2.4. Bone Processing and Histomorphometry

The harvested left femur was cleaned of soft tissues and sawed into halves. Then fixed with neutral buffered formalin for the decalcified section, and 70% alcohol for the undecalcified section. Using 10% ethylenediaminetetraacetic acid (EDTA) solution, the bone was then embedded in paraffin wax, sectioned at a thickness of 5 µm with a microtome (Leica RM2235, Nussloch, Germany), and stained with hematoxylin and eosin. The sections were subjected to the evaluation of static bone parameters. The static parameters measured included osteoblast surface (Ob.S/BS), osteoclast surface (Oc.S/BS), eroded surface (ES/BS), osteoid surface (OS/BS) and osteoid volume (OV/BV). For the undecalcified section, the bone was embedded in polymethyl methacrylate (Polysciences, PA, USA) and sectioned with a microtome into a thickness of 5 µm. The unstained sections were subjected to dynamic bone analysis. The dynamic parameters include single-labeled surface (sLS/BS), doubled-labeled surface (dLS/BS), mineralizing surface (MS/BS), mineral apposition rate (MAR), and bone formation rate (BFR). Whereas the sections stained with the von Kossa method were analyzed for structural bone indices, including bone volume/total volume (BV/TV), trabecular bone thickness (Tb.Th), trabecular bone number (Tb.N), and trabecular bone separation (Tb.Sp).

Micrographs of the bone sections were taken using a microscope (Nikon Eclipse 80i, Tokyo, Japan). The region of interest was the secondary spongiosa of the metaphysis located 1–3 mm distal to the growth plate. The assessment of bone static and dynamic histomorphometric parameters was performed using the Weibel Grid technique. The analysis of bone structural histomorphometric parameters was done using automated image analysis software (MediaCybernetics Image Pro-Plus, Rockville, MD, USA).

### 2.5. Bone Remodeling Markers and Hormones

The levels of serum osteocalcin (Immunodiagnostic Systems, Tyne and Wear, UK), carboxyl-terminal telopeptides of type 1 collagen (CTX-1) (Immunodiagnostic Systems, Tyne and Wear, UK), leptin (IBL International GmbH, Hamburg, Germany), adiponectin (Qayee Bio-Technology, Shanghai, China), and insulin (SPI-Bio, Bertin Pharma, Saclay, France) were measured using commercial rat-specific quantitative enzyme-linked immunosorbent assay kits, in accordance with the manufacturer’s instructions.

### 2.6. Statistical Analysis

Statistical analysis was performed using the Statistical Package for Social Sciences (SPSS) version 20 (IBM, Armonk, NY, USA). The fold change of MetS parameters was calculated as the final reading (week 20) over the baseline reading. The comparison of all parameters among the study groups was performed using one-way analysis of variance (ANOVA) with *Tukey’s post hoc* test. All data were presented as the mean ± SEM. A *p*-value of <0.05 was considered as statistically significant.

## 3. Results

**Figure 1:**
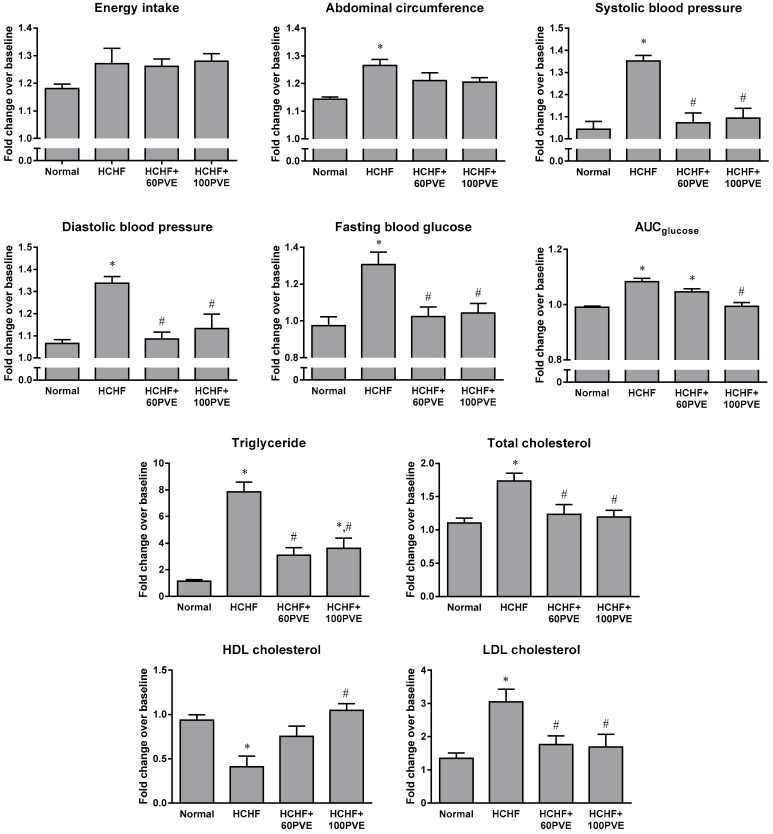
Fold changes in the MetS parameters of the normal, HCHF, HCHF+60PVE, and HCHF+100PVE animals, as compared to the baseline animals. The data are expressed as mean ± SEM. Letter ‘*’ indicates significant difference (*p* < 0.05) compared to the normal group and ‘#’ indicates significant difference (*p* < 0.05) compared to the HCHF group. Abbreviations: HCHF, high-carbohydrate high-fat; PVE, palm vitamin E.

The energy intake of animals between groups did not show significant change (*p* > 0.05). Animals fed with HCHF diet had higher abdominal circumference, systolic blood pressure, diastolic blood pressure, fasting blood glucose, glucose intolerance, triglyceride, total cholesterol, LDL cholesterol, fat mass, liver weight, lower HDL cholesterol, and showed no significant change in body weight compared to animals fed with standard rat chow (*p* < 0.05). Treatment with PVE at the dosage of 60 or 100 mg/kg significantly reduced blood pressure, fasting blood glucose, triglyceride, total cholesterol, and LDL cholesterol in the HCHF animals (*p* < 0.05). Only 100 mg/kg PVE improved the glucose tolerance and increased the level of HDL cholesterol in the HCHF rats (*p* < 0.05). However, there was no significant difference in abdominal circumference, body weight, fat mass, and liver weight of the PVE-treated animals, compared to the vehicle-treated animals (*p* > 0.05) (Table 1 and Figure 1).

Results obtained from DXA scan showed there was no significant difference in the whole body, femur, and tibia BMD among the normal and HCHF animals, with or without treatment of PVE (60 or 100 mg/kg) (*p* > 0.05) (Table 2). Static bone histomorphometric analysis demonstrated that the HCHF diet resulted in reductions in Ob.S/BS, OS/BS, OV/BV, and increment in ES/BS in animals when compared with the normal rats (*p* < 0.05). No significant change was observed in Oc.S/BS after feeding the animals with the HCHF diet (*p* > 0.05). Both 60 and 100 mg/kg PVE significantly increased Ob.S/BS and OV/BV, but only 100 mg/kg PVE reduced ES/BS in the HCHF rats (*p* < 0.05) (Table 3). Dynamic bone histomorphometric parameters were not significantly different among all the study groups (*p* > 0.05), with the exception that only 60 mg/kg PVE significantly reduced sLS/BS in animals provided with the HCHF diet (*p* < 0.05) (Table 3). For structural bone variables, the HCHF rats had significantly lower BV/TV (*p* < 0.05), but comparable Tb.Th, Tb.N and Tb.Sp, as compared to the normal rats (*p* > 0.05). Supplementation of PVE (60 or 100 mg/kg) improved BV/TV in the HCHF-fed animals (*p* < 0.05) (Table 3). For bone remodeling markers, the HCHF diet caused a significant increase in CTX-1 level (*p* < 0.05), but did not affect the osteocalcin level in male rats (*p* > 0.05). Oral administration of PVE had no effect on these bone remodeling markers (*p* > 0.05) (Table 4).

For hormonal changes, the HCHF diet caused elevations in leptin, insulin, and reduction in adiponectin levels in animal serums (*p* < 0.05). Treatment with PVE (60 or 100 mg/kg) significantly lowered the leptin level (*p* < 0.05), but did not cause any changes in the adiponectin and insulin levels of the HCHF animals (*p* > 0.05) (Table 5).

## 4. Discussion

Successful induction of MetS in male rats by the HCHF diet in the present study corroborated the findings of other researchers [38,39,40]. The HCHF diet utilized in this study was high in carbohydrate, fat, and salt contributing to the occurrence of abdominal obesity, hypertension, hyperglycemia, glucose intolerance, and dyslipidemia in animals. Our findings demonstrated that PVE normalized blood pressure, fasting blood glucose, and lipid profile in the HCHF rats. There was a dose-dependent effect of PVE in preventing medical conditions associated with MetS. Palm vitamin E at 100 mg/kg improved glucose tolerance and increased HDL cholesterol level in the HCHF rats. However positive effects were not seen in the HCHF animals treated with 60 mg/kg PVE. There was no change in abdominal circumference of the PVE-treated HCHF rats compared to the vehicle-treated HCHF rats. We assumed that this observation might be due to the animals having no physical activity or dietary restrictions. The beneficial effects of PVE in reversing the individual complication of MetS have been previously well-established [41]. Our current study further reported the advantages of PVE in preventing simultaneous MetS conditions.

Since MetS is associated with bone loss [42], we evaluated HCHF animal bone health with and without treatment of PVE, in terms of BMD, bone microstructure, remodeling event, and cellular properties. For DXA measurement, there was no significant difference in BMD on the animals whole body, femur, and tibia, among all the study groups. Previous animal studies indicated that the time needed to detect a significant change in BMD was 8 and 9 months in orchidectomized and ovariectomized rats, respectively [32,43]. Henceforth, a longer study period might be required to observe the changes in BMD of animals. However, previous human epidemiological studies reported no significant association between intake or serum level of vitamin E and BMD improvement [44,45,46,47]. The variables such as diet and physical activities were controlled in an animal experimental setting, whereas these confounding factors varied among individuals in day-to-day human life. We speculated that these differences contributed to the discrepancies in the effects of vitamin E on BMD in animal and human studies. Bone histomorphometric analysis found that the HCHF diet caused reductions in Ob.S/BS, OS/BS, OV/BV, and increase in ES/BS, subsequently leading to the reduction of BV/TV in animals. However, there was no effect of the HCHF diet on bone dynamic histomorphometric parameters. Apart from that, animals fed with 20 weeks of HCHF diet had significantly higher serum CTX-1 level, but no change in serum osteocalcin levels, when compared with rats fed with standard rat chow. Although the osteoclast numbers remained unchanged in the HCHF animals, the bone resorption activity was increased, indicated by the increase in the resorption cavities and serum CTX-1 level. Our findings also showed that the HCHF diet caused reductions in osteoblast proliferation and differentiation, leading to decreased bone matrix synthesis in the animals. Even though there was a reduction in osteoblast, the remaining osteoblast bone formation activity continued in response to the increased bone resorption activity. This explanation was evidenced in this study by the unchanged serum osteocalcin level and dynamic parameters of the HCHF animals.

Oral administration of PVE for 12 weeks significantly increased Ob.S/BS, OS/BS, as well as decreased ES/BS and sLS/BS in the HCHF animals. The structural parameters also demonstrated that BV/TV and Tb.Th in the HCHF animals increased after supplementation of PVE. Nevertheless, treatment with PVE did not affect BMD and bone remodeling markers in the HCHF rats compared with the vehicle-treated HCHF rats. In addition, we observed a lack of dose-dependent effects whereby the skeletal protecting properties of PVE at dosage levels of 60 mg/kg and 100 mg/kg were similar. Our findings were consistent with numerous previous studies. Ahmad et al. (2005) demonstrated that 100 mg/kg PVE increased osteoblast number, osteoid surface, and decreased eroded surface in ferric nitrilotriacetate-induced osteoporotic rats [33]. In a nicotine-induced osteoporotic rat model, 60 mg/kg PVE preserved the trabecular bone structure and decreased eroded surface [34]. In addition, the bone-protective effects of PVE were evaluated in ovariectomized [31,43] and orchidectomized rats [32].

In this study, we also investigated the involvement of hormones in regulating the bone metabolism in the experimental animals. The HCHF diet elevated levels of leptin, insulin, and reduced adiponectin level in the animal serum. The level of leptin is directly proportional to the fat intake, which has been proven in both human [48] and animal studies [49]. Adiponectin is also inversely correlated with the fat content in the diet. A previous study found that a high-fat diet increased leptin and reduced adiponectin levels in a mouse model of ovariectomy [50]. As for insulin, hyperglycemia is the stimulus for increased insulin secretion to enhance cell glucose uptake [51]. Our findings also indicated that PVE lowered the serum leptin level in the HCHF rats. Comparable in vivo studies on the effects of tocotrienol on leptin levels is limited. The level of adiponectin after treatment was not statistically different when compared to the normal and HCHF animals. Our result was consistent with a previous study using an obese mouse model induced by high-fat diet. The level of adiponectin in plasma was significantly decreased by HCHF diet, which was not changed by γ-tocotrienol supplementation (10 or 50 mg/kg) for 4 weeks [25]. This study indicated that even though the insulin level in PVE-treated animals remained high, the elevated blood glucose level was reversed. These observations implied the action of PVE in increasing insulin sensitivity. An in vitro study demonstrated that exposure of γ- and δ-tocotrienol to pancreatic β-cells from male Wistar rats revealed significantly higher quantitative expression in most of the insulin secretion-associated genes, suggesting the role of tocotrienol in regulating insulin synthesis [52]. Therefore, we assumed that in this study, the supplementation of PVE contributed to the increased insulin secretion in the HCHF rats, even though glucose tolerance was improved.

Overall, our findings suggested that the perturbation of leptin, adiponectin and insulin levels caused by the HCHF diet could contribute to the imbalance of bone formation and resorption activities. This favored a net bone loss at the trabecular regions in male rats. Treatment with PVE prevented most of the medical complications associated to MetS and normalized leptin level in animals with MetS induced HCHF diet, which might be, in part, resulting in the bone-protective properties of PVE. The relationship between leptin and bone is complex with previous studies producing conflicting data. Ducy et al. found that leptin-deficient mice are obese and had increased bone formation, leading to high bone mass. Intracerebroventricular administration of leptin caused bone loss in these leptin-deficient mice [17]. Consistent with these results, later investigation also showed that mice with hyperleptinemia displayed low bone BV/TV [53]. Conversely, Turner et al. reported that leptin increased osteoblast number and activity, acting through peripheral pathways [54]. Taken together, these previous findings suggested that leptin-regulated bone mass differ throughout the central and sympathetic nervous system. Nonetheless, the relative contribution of leptin on bone metabolism remains to be resolved, owing to the dual controls of leptin.

We also addressed several limitations of this study. Firstly, skeletally immature (12-week-old) rats were used and it is therefore debatable whether MetS causes bone loss or stunted bone growth. Our earlier data from sequential micro-computed tomography scans indicated the occurrence of bone loss attributed to MetS induced by HCHF diet, evidenced by the gradual degradation of bone microstructure from week 0 to week 20 [5]. Secondly, other histological staining such as tartrate-resistance acid phosphatase (TRAP) was not performed. Thirdly, the liver toxicity assessment [such as the measurement of aspartate transaminase (AST), alanine transaminase (ALT), and alkaline phosphatase (ALP) levels] was not conducted. However, the study by Nakamura et al. indicated that the no-observed-adverse-effect level (NOAEL) for PVE is 120 mg/kg body weight per day for male rats [55]. Therefore, it is assumed that treatment with PVE might not cause any adverse effect to liver function in animals as the doses were lower than 120 mg/kg in this study. Fourthly, other possible underlying mechanisms that closely link MetS and bone loss, such as the oxidative status, inflammatory response, and expression of OPG/RANKL were not investigated. The PVE used was a mixture of four isomers of tocotrienol and α-tocopherol. It is impossible to delineate the activity of each compound, thus the most active compound responsible for reversing MetS and osteoporosis awaits further experimentation in both animals and humans. We speculated the involvement of mevalonate pathway suppression and anti-inflammation as possible pathways contributing to the effects of PVE on MetS and bone.

## 5. Conclusions

In summary, PVE reverses most of the features of MetS, except for obesity-related parameters. Besides, PVE exerts anabolic and anti-resorptive effects on bone. These effects may be, in part, attributed to the normalization of leptin levels in male rats. This study is the first report evaluating the effects of PVE in the regulation of hormonal change during MetS and osteoporosis, using a rat model of bone loss due to MetS. Further investigations from a human clinical trial are warranted to further validate these results.

## Figures and Tables

**Table 1:** The measurement of body weight, fat mass, and liver weight in all the experimental groups at the end of the study.

	Baseline	Normal	HCHF	HCHF + 60 PVE	HCHF + 100 PVE
Body weight (g)	225.50 ± 6.22	374.67 ± 3.61 *^a^*	372.17 ± 10.84 *^a^*	361.67 ± 13.11 *^a^*	348.67 ± 11.36 *^a^*
Fat mass (g)	11.83 ± 2.32	26.35 ± 4.11	58.57 ± 5.00 *^a^*^,^*	56.45 ± 3.53 *^a^*^,^*	61.42 ± 2.91 *^a^*^,^*
Liver weight (g)	9.88 ± 0.36	11.81 ± 0.18	14.81 ± 0.73 *^a^*^,^*	12.85 ± 0.51 *^a^*	12.78 ± 0.86 *^a^*

The data are expressed as mean ± SEM. Letter ‘*^a^*’ indicates significant difference (*p* < 0.05) compared to the baseline group. Symbol ‘*’ indicates significant difference (*p* < 0.05) compared to the normal group. Abbreviations: HCHF, high-carbohydrate high-fat; PVE, palm vitamin E.

**Table 2:** The measurement of whole body, femur, and tibia BMD in all the experimental groups at the end of the study.

	Baseline	Normal	HCHF	HCHF + 60 PVE	HCHF + 100 PVE
Whole body BMD (g/cm^2^)	0.129 ± 0.001	0.182 ± 0.003 *^a^*	0.182 ± 0.005 *^a^*	0.186 ± 0.005 *^a^*	0.186 ± 0.002 *^a^*
Left femur BMD (g/cm^2^)	0.228 ± 0.002	0.304 ± 0.006 *^a^*	0.306 ± 0.007 *^a^*	0.313 ± 0.007 *^a^*	0.299 ± 0.007 *^a^*
Right femur BMD (g/cm^2^)	0.230 ± 0.005	0.283 ± 0.007 *^a^*	0.287 ± 0.012 *^a^*	0.295 ± 0.010 *^a^*	0.302 ± 0.008 *^a^*
Left tibia BMD (g/cm^2^)	0.159 ± 0.003	0.207 ± 0.003 *^a^*	0.215 ± 0.005 *^a^*	0.219 ± 0.002 *^a^*	0.202 ± 0.009 *^a^*
Right tibia BMD (g/cm^2^)	0.158 ± 0.002	0.185 ± 0.009	0.190 ± 0.013	0.197 ± 0.010	0.197 ± 0.012

The data are expressed as mean ± SEM. Letter ‘*^a^*’ indicates significant difference (*p* < 0.05) compared to the baseline group. Abbreviations: HCHF, high-carbohydrate high-fat; PVE, palm vitamin E.

**Table 3:** The measurement of bone static (Ob.S/BS, Oc.S/BS, ES/BS, OS/BS, and OV/BV), dynamic (sLS/BS, dLS/BS, MS/BS, MAR, and BFR), and structural (BV/TV, Tb.Th, Tb.N, and Tb.Sp) indices in all the experimental groups at the end of the study.

	Baseline	Normal	HCHF	HCHF + 60 PVE	HCHF + 100 PVE
Ob.S/BS (%)	5.22 ± 0.77	8.67 ± 0.58 *^a^*	4.28 ± 0.30 *	8.58 ± 0.88 *^a^*^,#^	8.38 ± 0.92 *^a^*^,#^
Oc.S/BS (%)	4.08 ± 0.64	5.74 ± 0.93	4.71 ± 0.53	5.25 ± 0.22	4.22 ± 0.35
ES/BS (%)	7.47 ± 0.62	6.07 ± 0.73	12.04 ± 1.37 *^a^*^,^*	9.49 ± 0.68*	8.24 ± 0.31^#^
OS/BS (%)	5.76 ± 1.01	9.75 ± 0.72 *^a^*	4.72 ± 0.47*	8.26 ± 0.32^#^	8.46 ± 0.16 *^a^*^,#^
OV/BV (%)	4.70 ± 0.36	7.88 ± 0.86 *^a^*	3.47 ± 0.63*	4.60 ± 0.31*	4.54 ± 0.69*
sLS/BS (%)	11.34 ± 1.01	11.76 ± 0.79	13.76 ± 0.77	7.82 ± 1.05*^,#^	9.98 ± 1.00
dLS/BS (%)	10.65 ± 0.83	10.44 ± 0.86	9.13 ± 0.53	11.51 ± 0.98	8.20 ± 1.23
MS/BS (%)	17.62 ± 0.89	17.11 ± 0.71	16.73 ± 0.69	14.19 ± 0.61 *^a^*	15.21 ± 0.64
MAR (µm/day)	1.60 ± 0.07	1.65 ± 0.19	1.49 ± 0.08	1.78 ± 0.14	1.42 ± 0.13
BFR (µm^3^/µm^2^/day)	25.45 ± 1.77	23.92 ± 1.81	21.37 ± 1.41	23.53 ± 1.63	21.52 ± 2.24
BV/TV (%)	32.09 ± 2.56	29.78 ± 2.30	18.98 ± 0.90 *^a^*^,^*	32.55 ± 1.36^#^	30.90 ± 0.63 ^#^
Tb.Th (µm)	108.20 ± 6.95	92.68 ± 4.86	70.28 ± 13.00 *^a^*	101.44 ± 9.46	99.44 ± 6.72
Tb.N (µm^−1^)	0.0030 ± 0.0003	0.0032 ± 0.0002	0.0033 ± 0.0007	0.0034 ± 0.0001	0.0032 ± 0.0002
Tb.Sp (µm)	250.47 ± 29.35	226.24 ± 17.24	313.71 ± 60.97	207.64 ± 8.73	222.95 ± 13.43

The data are expressed as mean ± SEM. Letter ‘*^a^*’ indicates significant difference (*p* < 0.05) compared to the baseline group. Symbol ‘*’ indicates significant difference (*p* < 0.05) compared to the normal group, and ‘^#^’ indicates significant difference (*p* < 0.05) compared to the HCHF group. Abbreviations: HCHF, high-carbohydrate high-fat; PVE, palm vitamin E.

**Table 4:** The measurement of bone remodeling markers (osteocalcin and CTX-1) in all the experimental groups at the end of the study.

	Baseline	Normal	HCHF	HCHF + 60 PVE	HCHF + 100 PVE
Osteocalcin (ng/mL)	773.28 ± 38.01	401.94 ± 13.34 *^a^*	305.55 ± 15.65 *^a^*	386.49 ± 25.73 *^a^*	369.19 ± 22.15 *^a^*
CTX-1 (ng/mL)	42.47 ± 3.13	17.87 ± 0.36 *^a^*	24.48 ± 1.44 *^a^*^,^*	19.22 ± 0.69 *^a^*	20.85 ± 1.22 *^a^*

The data are expressed as mean ± SEM. Letter ‘*^a^*’ indicates significant difference (*p* < 0.05) compared to the baseline group. Symbol ‘*’ indicates significant difference (*p* < 0.05) compared to the normal group. Abbreviations: HCHF, high-carbohydrate high-fat; PVE, palm vitamin E.

**Table 5:** The measurement of hormone levels (leptin, adiponectin, and insulin) in all the experimental groups at the end of the study.

	Baseline	Normal	HCHF	HCHF + 60 PVE	HCHF + 100 PVE
Leptin (pg/mL)	530.36 ± 74.14	1134.70 ± 153.36	3425.55 ± 377.31 *^a^*^,^*	2050.00 ± 246.92 *^a^*^,^*^,#^	530.36 ± 74.14
Adiponectin (ng/mL)	59.11 ± 1.29	58.51 ± 1.76	50.87 ± 1.43*	56.55 ± 2.58	59.11 ± 1.29
Insulin (ng/mL)	0.35 ± 0.04	0.38 ± 0.02	6.51 ± 0.55 *^a^*^,^*	7.62 ± 0.50 *^a^*^,^*	0.35 ± 0.04

The data are expressed as mean ± SEM. Letter ‘*^a^*’ indicates significant difference (*p* < 0.05) compared to the baseline group. Symbol ‘*’ indicates significant difference (*p* < 0.05) compared to the normal group, and ‘^#^’ indicates significant difference (*p* < 0.05) compared to the HCHF group. Abbreviations: HCHF, high-carbohydrate high-fat; PVE, palm vitamin E.

## References

[1] Alberti K.G., Eckel R.H., Grundy S.M., Zimmet P.Z., Cleeman J.I., Donato K.A., Fruchart J.C., James W.P., Loria C.M., Smith S.C. (2009). Harmonizing the metabolic syndrome: A joint interim statement of the International Diabetes Federation Task Force on Epidemiology and Prevention; National Heart, Lung, and Blood Institute; American Heart Association; World Heart Federation; International Atherosclerosis Society; and International Association for the Study of Obesity. Circulation.

[2] Lee K. (2015). Metabolic Syndrome and Osteoporosis in Relation to Muscle Mass. Calcif. Tissue Int..

[3] Hwang D.K., Choi H.J. (2010). The relationship between low bone mass and metabolic syndrome in Korean women. Osteoporos. Int..

[4] Kim H.Y., Choe J.W., Kim H.K., Bae S.J., Kim B.J., Lee S.H., Koh J.M., Han K.O., Park H.M., Kim G.S. (2010). Negative association between metabolic syndrome and bone mineral density in Koreans, especially in men. Calcif. Tissue Int..

[5] Wong S.K., Chin K.Y., Suhaimi F.H., Ahmad F., Jamil N.A., Ima-Nirwana S. (2017). Osteoporosis is associated with metabolic syndrome induced by high-carbohydrate high-fat diet in a rat model. Biomed. Pharmacother..

[6] Wong S.K., Chin K.-Y., Suhaimi F.H., Ahmad F., Ima-Nirwana S. (2018). Effects of metabolic syndrome on bone mineral density, histomorphometry and remodelling markers in male rats. PLoS ONE.

[7] Sekar S., Shafie S.R., Prasadam I., Crawford R., Panchal S.K., Brown L., Xiao Y. (2017). Saturated fatty acids induce development of both metabolic syndrome and osteoarthritis in rats. Sci. Rep..

[8] Sun A.R., Panchal S.K., Friis T., Sekar S., Crawford R., Brown L., Xiao Y., Prasadam I. (2017). Obesity-associated metabolic syndrome spontaneously induces infiltration of pro-inflammatory macrophage in synovium and promotes osteoarthritis. PLoS ONE.

[9] Hukshorn C.J., Saris W.H. (2004). Leptin and energy expenditure. Curr. Opin. Clin. Nutr. Metab. Care.

[10] Qi Y., Takahashi N., Hileman S.M., Patel H.R., Berg A.H., Pajvani U.B., Scherer P.E., Ahima R.S. (2004). Adiponectin acts in the brain to decrease body weight. Nat. Med..

[11] Yamauchi T., Kamon J., Waki H., Terauchi Y., Kubota N., Hara K., Mori Y., Ide T., Murakami K., Tsuboyama-Kasaoka N. (2001). The fat-derived hormone adiponectin reverses insulin resistance associated with both lipoatrophy and obesity. Nat. Med..

[12] Ye J. (2013). Mechanisms of insulin resistance in obesity. Front. Med..

[13] Iwamoto I., Fujino T., Douchi T. (2004). The leptin receptor in human osteoblasts and the direct effect of leptin on bone metabolism. Gynecol. Endocrinol..

[14] Lin Y.Y., Chen C.Y., Chuang T.Y., Lin Y., Liu H.Y., Mersmann H.J., Wu S.C., Ding S.T. (2014). Adiponectin receptor 1 regulates bone formation and osteoblast differentiation by GSK-3beta/beta-catenin signaling in mice. Bone.

[15] Vianna A.G.D., Sanches C.P., Barreto F.C. (2017). Review article: Effects of type 2 diabetes therapies on bone metabolism. Diabetol. Metab. Syndr..

[16] Upadhyay J., Farr O.M., Mantzoros C.S. (2015). The role of leptin in regulating bone metabolism. Metabolism.

[17] Ducy P., Amling M., Takeda S., Priemel M., Schilling A.F., Beil F.T., Shen J., Vinson C., Rueger J.M., Karsenty G. (2000). Leptin inhibits bone formation through a hypothalamic relay: A central control of bone mass. Cell.

[18] Iwase S., Nishimura N., Mano T., Flores M.V. (2013). Osteoporosis in Spaceflight. Topics in Osteoporosis.

[19] Lubkowska A., Dobek A., Mieszkowski J., Garczynski W., Chlubek D. (2014). Adiponectin as a Biomarker of Osteoporosis in Postmenopausal Women: Controversies. Dis. Mark..

[20] Karsenty G., Ferron M. (2012). The contribution of bone to whole-organism physiology. Nature.

[21] Kaur J. (2014). A Comprehensive Review on Metabolic Syndrome. Cardiol. Res. Pract..

[22] Kling J.M., Clarke B.L., Sandhu N.P. (2014). Osteoporosis Prevention, Screening, and Treatment: A Review. J. Women’s Health.

[23] Wong W.Y., Poudyal H., Ward L.C., Brown L. (2012). Tocotrienols reverse cardiovascular, metabolic and liver changes in high carbohydrate, high fat diet-fed rats. Nutrients.

[24] Wong W.Y., Ward L.C., Fong C.W., Yap W.N., Brown L. (2015). Anti-inflammatory gamma- and delta-tocotrienols improve cardiovascular, liver and metabolic function in diet-induced obese rats. Eur. J. Nutr..

[25] Zhao L., Kang I., Fang X., Wang W., Lee M.A., Hollins R.R., Marshall M.R., Chung S. (2015). Gamma-tocotrienol attenuates high-fat diet-induced obesity and insulin resistance by inhibiting adipose inflammation and M1 macrophage recruitment. Int. J. Obes..

[26] Siddiqui S., Rashid Khan M., Siddiqui W.A. (2010). Comparative hypoglycemic and nephroprotective effects of tocotrienol rich fraction (TRF) from palm oil and rice bran oil against hyperglycemia induced nephropathy in type 1 diabetic rats. Chem. Biol. Interact..

[27] Newaz M.A., Yousefipour Z., Nawal N., Adeeb N. (2003). Nitric oxide synthase activity in blood vessels of spontaneously hypertensive rats: Antioxidant protection by gamma-tocotrienol. J. Physiol. Pharmacol..

[28] Qureshi A.A., Peterson D.M., Hasler-Rapacz J.O., Rapacz J. (2001). Novel tocotrienols of rice bran suppress cholesterogenesis in hereditary hypercholesterolemic swine. J. Nutr..

[29] Kuhad A., Chopra K. (2009). Attenuation of diabetic nephropathy by tocotrienol: Involvement of NFkB signaling pathway. Life Sci..

[30] Kuhad A., Bishnoi M., Tiwari V., Chopra K. (2009). Suppression of NF-kappabeta signaling pathway by tocotrienol can prevent diabetes associated cognitive deficits. Pharmacol. Biochem. Behav..

[31] Soelaiman I.N., Ming W., Abu Bakar R., Hashnan N.A., Mohd Ali H., Mohamed N., Muhammad N., Shuid A.N. (2012). Palm tocotrienol supplementation enhanced bone formation in oestrogen-deficient rats. Int. J. Endocrinol..

[32] Ima-Nirwana S., Kiftiah A., Zainal A., Norazlina M., Gapor M., Khalid B. (2000). Palm vitamin E prevents osteoporosis in orchidectomized growing male rats. Nat. Prod. Sci..

[33] Ahmad N.S., Khalid B.A., Luke D.A., Ima Nirwana S. (2005). Tocotrienol offers better protection than tocopherol from free radical-induced damage of rat bone. Clin. Exp. Pharmacol. Physiol..

[34] Hermizi H., Faizah O., Ima-Nirwana S., Ahmad Nazrun S., Norazlina M. (2009). Beneficial effects of tocotrienol and tocopherol on bone histomorphometric parameters in sprague-dawley male rats after nicotine cessation. Calcif. Tissue Int..

[35] Wong S.K., Chin K.-Y., Suhaimi F.H., Ahmad F., Ima-Nirwana S. (2018). The effects of palm tocotrienol on metabolic syndrome and bone loss in male rats induced by high-carbohydrate high-fat diet. J. Funct. Foods..

[36] Wong S.K., Chin K.-Y., Suhaimi F.H., Ahmad F., Ima-Nirwana S. (2018). Exploring the potential of tocotrienol from *Bixa. orellana.* as a single agent targeting metabolic syndrome and bone loss. Bone.

[37] Wong S.K., Chin K.Y., Suhaimi F.H., Ahmad F., Ima-Nirwana S. (2018). The effects of a modified high-carbohydrate high-fat diet on metabolic syndrome parameters in male rats. Exp. Clin. Endocrinol. Diabetes.

[38] Poudyal H., Panchal S., Brown L. (2010). Comparison of purple carrot juice and beta-carotene in a high-carbohydrate, high-fat diet-fed rat model of the metabolic syndrome. Br. J. Nutr..

[39] Hao L., Lu X., Sun M., Li K., Shen L., Wu T. (2015). Protective effects of L-arabinose in high-carbohydrate, high-fat diet-induced metabolic syndrome in rats. Food Nutr. Res..

[40] Panchal S.K., Poudyal H., Iyer A., Nazer R., Alam A., Diwan V., Kauter K., Sernia C., Campbell F., Ward L. (2011). High-carbohydrate high-fat diet-induced metabolic syndrome and cardiovascular remodeling in rats. J. Cardiovasc. Pharmacol..

[41] Wong S.K., Chin K.Y., Suhaimi F.H., Ahmad F., Ima-Nirwana S. (2017). Vitamin E as a Potential Interventional Treatment for Metabolic Syndrome: Evidence from Animal and Human Studies. Front. Pharmacol..

[42] Wong S.K., Chin K.Y., Suhaimi F.H., Ahmad F., Ima-Nirwana S. (2016). The Relationship between Metabolic Syndrome and Osteoporosis: A Review. Nutrients.

[43] Norazlina M., Ima-Nirwana S., Gapor M.T., Khalid B.A. (2000). Palm vitamin E is comparable to alpha-tocopherol in maintaining bone mineral density in ovariectomised female rats. Exp. Clin. Endocrinol. Diabetes.

[44] Macdonald H.M., New S.A., Golden M.H., Campbell M.K., Reid D.M. (2004). Nutritional associations with bone loss during the menopausal transition: Evidence of a beneficial effect of calcium, alcohol, and fruit and vegetable nutrients and of a detrimental effect of fatty acids. Am. J. Clin. Nutr..

[45] Maggio D., Barabani M., Pierandrei M., Polidori M.C., Catani M., Mecocci P., Senin U., Pacifici R., Cherubini A. (2003). Marked decrease in plasma antioxidants in aged osteoporotic women: Results of a cross-sectional study. J. Clin. Endocrinol. Metab..

[46] Wolf R.L., Cauley J.A., Pettinger M., Jackson R., Lacroix A., Leboff M.S., Lewis C.E., Nevitt M.C., Simon J.A., Stone K.L. (2005). Lack of a relation between vitamin and mineral antioxidants and bone mineral density: Results from the Women’s Health Initiative. Am. J. Clin. Nutr..

[47] Chin K.Y., Ima-Nirwana S. (2014). The effects of alpha-tocopherol on bone: A double-edged sword?. Nutrients.

[48] Rojo-Martinez G., Soriguer F.J., Gonzalez-Romero S., Tinahones F., Moreno F., de Adana S.R., Garriga M.J., Esteva I., Garcia-Arnes J., Gomez-Zumaquero J.M. (2000). Serum leptin and habitual fatty acid dietary intake in patients with type 1 diabetes mellitus. Eur. J. Endocrinol..

[49] Handjieva-Darlenska T., Boyadjieva N. (2009). The effect of high-fat diet on plasma ghrelin and leptin levels in rats. J. Physiol. Biochem..

[50] Ludgero-Correia A., Aguila M.B., Mandarim-de-Lacerda C.A., Faria T.S. (2012). Effects of high-fat diet on plasma lipids, adiposity, and inflammatory markers in ovariectomized C57BL/6 mice. Nutrition.

[51] Saltiel A.R., Kahn C.R. (2001). Insulin signalling and the regulation of glucose and lipid metabolism. Nature.

[52] Chia L.L., Jantan I., Chua K.H., Lam K.W., Rullah K., Aluwi M.F.M. (2016). Effects of Tocotrienols on Insulin Secretion-Associated Genes Expression of Rat Pancreatic Islets in a Dynamic Culture. Front. Pharmacol..

[53] Elefteriou F., Takeda S., Ebihara K., Magre J., Patano N., Ae Kim C., Ogawa Y., Liu X., Ware S.M., Craigen W.J. (2004). Serum leptin level is a regulator of bone mass. Proc. Natl. Acad. Sci. USA.

[54] Turner R.T., Kalra S.P., Wong C.P., Philbrick K.A., Lindenmaier L.B., Boghossian S., Iwaniec U.T. (2013). Peripheral leptin regulates bone formation. J. Bone Miner. Res..

[55] Nakamura H., Furukawa F., Nishikawa A., Miyauchi M., Son H.Y., Imazawa T., Hirose M. (2001). Oral toxicity of a tocotrienol preparation in rats. Food Chem. Toxicol..

